# Calcitonin-Gene-Related Peptide in Migraine and Tension-Type Headache in Children During Interictal Period

**DOI:** 10.3390/diagnostics14232645

**Published:** 2024-11-24

**Authors:** Jadranka Sekelj Fures, Vlasta Duranovic, Jasna Lenicek Krleza, Ana Katusic Bojanac, Lana Loncar, Ivana Dakovic, Sanja Pejic-Rosko, Katarina Vulin, Andrijana Pilon-Far, Andrea Simic Klaric

**Affiliations:** 1 Department of Pediatric Neurology, Children’s Hospital Zagreb, 10000 Zagreb, Croatia; 2 Faculty of Medicine Osijek, Josip Juraj Strossmayer University of Osijek, 31000 Osijek, Croatia; 3 Department of Laboratory Diagnostics, Children’s Hospital Zagreb, 10000 Zagreb, Croatia; 4 University Department of Nursing, Catholic University of Croatia, Ilica 244, 10000 Zagreb, Croatia; 5 Department of Laboratory Medical Diagnostics, University of Applied Health Sciences Zagreb, 10000 Zagreb, Croatia; 6 Department of Medical Biology, School of Medicine, University of Zagreb, 10000 Zagreb, Croatia; 7 Centre of Excellence for Reproductive and Regenerative Medicine, School of Medicine, University of Zagreb, 10000 Zagreb, Croatia; 8 Department of Medical and Laboratory Genetics, Children’s Hospital Zagreb, 10000 Zagreb, Croatia; 9 County General Hospital Požega, 34000 Požega, Croatia

**Keywords:** pediatric migraine, pediatric tension-type headache, CGRP

## Abstract

**Background/Objectives:** Research on calcitonin-gene-related peptide (CGRP) in adult migraine is extensive, but its role in childhood migraine remains unclear. This study aimed to evaluate serum CGRP levels in children experiencing migraine and tension-type headache (TTH) during interictal periods, comparing these levels to age-matched healthy controls. **Methods:** A total of 66 migraine patients, 59 with TTH, and 53 controls were recruited and stratified by headache onset age: under 7, 7–12, and over 12 years. CGRP levels were quantified using enzyme-linked immunosorbent assay (ELISA). **Results:** The migraine patients showed significantly higher serum CGRP levels than both the TTH patients and the controls (*p* < 0.001), with no significant difference between the latter two groups. Among the migraine patients, those without aura (MO) exhibited higher CGRP levels than those with aura (MA). The CGRP levels were lower in the. MA patients whose headaches began between ages 7 and 12 compared to the subjects with MO, while no significant differences were found in the patients whose headaches began after age 12. **Conclusions:** These findings suggest that elevated serum CGRP is indicative of pediatric migraine, with variations based on migraine type and age of onset. The difference in CGRP in preadolescent migraineurs with and without aura suggest that CGRP levels may vary depending on age and on migraine type.

## 1. Introduction

Headache is the most common cause of chronic or recurrent pain in childhood and adolescence [1] and affects school performance, social and physical activities, and quality of life [2,3], but it is still underdiagnosed and insufficiently treated. The longer course of headache means greater risk of developing chronic headache and associated comorbidities, such as anxiety and depression [4,5]. Therefore, it is essential to make an early diagnosis and implement preventive measures.

The most common primary headaches in children are migraine and tension-type headache [6]. These two types of headaches have a shared prevalence of 62% [7]. A recent meta-analysis revealed that the prevalence of childhood migraine is 11% [7], indicating an increase compared to previously known data. The prevalence of tension-type headache is from 17% to 29% [7,8], emphasizing that the prevalence of tension-type headache is more diverse due to the variability of headache characteristics during childhood and adolescence [9].

Migraine and tension-type headache (TTH) profoundly affect both individuals and society, particularly since these conditions impact people at a younger age, a time dedicated to developing academic and social skills. According to the 2019 Global Burden of Disease (GBD) findings, headache disorders rank among the top ten causes of disability-adjusted life years (DALYs) for individuals aged 10 to 49. The global incidence of migraine in this age group has increased by 16% since 1990, alongside a 37% rise in TTH incidence [10]. Data from 2021 indicate an even more pronounced increase in worldwide prevalence over a two-year period [11]. The prevalence, incidence, and corresponding effects of headaches on daily life vary across Sociodemographic Index (SDI) regions; however, a global school-based study led by the Global Campaign against Headache has successfully collected data from countries that previously lacked information on the epidemiology of primary headaches in children. Such studies are commendable as they enhance awareness of the condition’s prevalence and are crucial for informing educational and health policies in those nations [12,13,14,15].

The diagnosis of migraine and tension-type headache is based on the criteria of the International Classification of Headache disorders, third edition (ICHD-3), and these criteria are applicable in both children and adults, with some differences [16]. Some of the differences are officially included in ICHD-3, such as the duration of migraine attacks being 2–72 h in children, while others are only mentioned in the commentary in ICHD-3. [16]. One of the main controversies in clinical work is the duration of migraine pain in children, with studies showing that migraine pain in children can last less than 1 h [17,18,19,20]. Another difference is the localization of the migraine headache, as the pain in children is often bilateral, while unilateral pain occurs from adolescence [20,21,22,23]. The character of the pain also differs, with throbbing pain being most common in children [24], and pulsating pain most common in adults. Considering these differences, migraine and tension type-headache have more overlapping than distinctive features, making it even more difficult to differentiate the type of primary headache based solely on clinical characteristics.

One clear distinguishing factor between the two most common types of primary headaches is their pathophysiology. The pathophysiology of tension-type headache is the result of a combination of genetic factors, myofascial mechanisms, and central sensitization of nociceptive pathways, as well as dysfunction in the descending modulation of pain, which leads to the development of chronic pain [25]. Migraine, on the other hand, has several phases, including the prodromal phase, aura phase, headache phase, postdrome phase, and interictal phase, each characterized by specific pathophysiological hallmarks [26]. The prodromal phase can start hours or even days before the headache phase and involves a complex interaction between cortical and subcortical brain regions, with the hypothalamus as the main driver [27]. The aura phase is specific to migraine with aura and is associated with cortical spreading depression (CSD), representing a slowly spreading wave of depolarization followed by hyperpolarization of cortical neuronal and glial cells [28,29].

CSD activates the trigeminovascular system (TGVS), initiating the headache phase [30,31]. TGVS activation causes the release of various neuropeptides in the vascular branches of the meninges, including substance P (SP), pituitary adenylate cyclase-activating polypeptide (PACP), neurokinin A, and calcitonin-gene-related peptide (CGRP)—a neuropeptide that plays a crucial role in migraine [32,33,34,35,36,37]. The postdrome phase occurs between the end of the headache phase and the return to the state before the onset of the migraine attack. Symptoms during this phase may include neck stiffness, difficulty concentrating, and fatigue, lasting for more than 24 h [38,39,40]. The interictal phase is the period between two migraine attacks.

Although migraine and tension-type headache (TTH) are associated with the trigeminovascular system in their underlying mechanisms, research involving the adult population has consistently shown that patients with TTH maintain normal CGRP levels in cranial and peripheral blood, as well as in cerebrospinal fluid, which do not elevate during spontaneous or provoked headache episodes [41,42,43]. In addition to CGRP, plasma levels of other neuropeptides possibily involved in migraine patophysiology were normal in the cranial and peripheral blood of patients with TTH, irrespective of headache status [43,44].

CGRP acts on multiple sites along the trigeminovascular pathway. It is a potent vasodilator of cerebral blood vessels [45,46,47,48], involved in initiating neurogenic inflammation [49], and mediating peripheral and central sensitization [36,50,51,52]. Numerous studies confirm the central role of CGRP in the pathophysiology of migraine in adults [53,54,55,56,57,58,59,60].

The potential importance of CGRP in childhood migraine pathology has been explored in a small number of studies with inconsistent results. A study comparing the levels of CGRP in the blood during (ictally) and between migraine attacks (interictally) in adolescent subjects (aged 13–18 years) with migraine without aura (MO) and migraine with aura (MA) compared to healthy controls found no difference in CGRP levels between the study groups during non-attack periods. However, higher levels of CGRP were reported during attacks in subjects with MA [61]. Fan et al. found higher ictal and interictal levels of CGRP in migraineurs compared to healthy controls and a group with non-migraine headache [62,63]. Similarly, Liu et al. demonstrated higher plasma CGRP levels in children with migraine compared to healthy controls. Furthermore, CGRP levels were found to be higher during ictal periods than interictal periods, and they were also higher in subjects with MA compared to MO subjects [64]. In contrast to these studies, Hanci et al. found no differences in CGRP levels between children with migraine and healthy controls, both ictally and interictally [65]. The inconsistency and lack of reproducibility of the performed studies are probably the result of the different methodologies for measuring plasma CGRP, as well as the inhomogeneity of the study subjects [66].

This study prospectively investigated interictal serum CGRP levels in children with migraine compared to children with tension-type headache and healthy controls. The results were analyzed based on different subgroups of patients, and the correlation between CGRP levels and the demographic and clinical characteristics of the subjects was studied. The diagnostic value of CGRP in children with different types of primary headaches was also analyzed.

## 2. Materials and Methods

### 2.1. Study Participants

We prospectively enrolled 178 study subjects who attended the pediatric neurology clinic at the Children’s Hospital Zagreb, a tertiary medical center in Croatia. Among them, 66 (37.1%) had migraine, 59 (33.1%) had TTH, and 53 (29.8%) were healthy controls. Participants were grouped based on the age of headache onset: <7 years, 7–12 years, and >12 years.

Inclusion criteria for migraine patients included diagnosis of MO or MA or probable migraine with or without aura (pMA, pMO) defined by ICHD-3 [10]. Tension-type headache group included subjects with episodic tension-type headache (TTH) and probable episodic tension-type headache (pTTH), also defined by the ICHD-3 [10]. In the tension-type headache group, 13 participants (22%) had pTTH and 46 (78%) had definitive TTH, in the migraine group, 40 (60%) subjects had MO and 12 (18%) subjects had MA, and the pMA/pMO type was experienced by 7 (11%) respondents in each subgroup. The exclusion criteria were secondary headache, headache after head trauma, other primary headaches except migraine and tension-type headache, chronic migraine or chronic tension-type headache, prophylactic headache therapy, migraine attack or episode of tension-type headache within 72 h before blood sampling for CGRP, abortive headache therapy taken within 72 h before blood sampling for CGRP, chronic inflammatory and autoimmune diseases, mental retardation, and congenital or chromosomal anomalies.

The control group consisted of healthy children whose blood was sampled due to preoperative work-up or anesthesiologic procedures, and who had no anamnestic data for migraine, headache, and chronic inflammatory or autoimmune diseases.

All participants underwent a general physical and neurological examination, and diagnoses were reevaluated after two years. Those in the probable migraine/pTTH subgroups were re-assessed by another pediatric neurologist.

This study adhered to the Declaration of Helsinki and received ethical approval from relevant committees, with informed consent obtained from parents/guardians and study participants older than 9 years.

### 2.2. Demographic and Clinical Characteristics

Data collected included age, sex, family history of migraine, and previous serious illnesses. Major migraine symptoms recorded encompassed age of onset, course of headache in months, headache frequency, characteristics (duration, location, pain type), intensity assessed via Visual Analogue Scale (VAS), association of headache with usual physical activity, presence of nausea, vomiting, photophobia, phonophobia, and existence of and type of aura. These same features were collected for TTH subjects.

Headache course was computed from the first attack until blood sampling, duration was defined as the mean duration per attack per subject, while intensity was assessed based on the VAS for children over 7 years, using parental input for younger children. Headache frequency indicated monthly attack counts.

### 2.3. CGRP Determination

Blood for CGRP sampling was collected during venipuncture at the Children’s Hospital Zagreb, from the right or left cubital vein, with the patient in a lying position. A total of 3.5 mL of blood was collected into vacuum tubes (Vacuette, gold-yellow cap, 3.5 mL with gel separator and clot activator, Grainer Bio-One GmbH, Kremsmünster, Austria).

After collection, the blood sample was immediately centrifuged (10 min at 3500 rpm; centrifuge: Hettich GmbH Rotofix 32 A, Andreas Hettich GmbH & Co.KG, Kirchlengern, Germany, 2017). The serum sample was separated after routine laboratory analyses (minimum volume 300 µL) into plastic tubes with a cap and stored at −80 °C (TSE240VGP—ULT freezer, −86 °C, Thermo Scientific Waltham, MA, USA, 2015) until the CGRP determination. The determination of CGRP was performed using the ELISA method according to the manufacturer’s instructions [67] on a VersaMax microplate reader (Molecular Devices Corporation, Orleans Drive, Sunnyvale, CA, USA, 2017) at a wavelength of 405 nm, with results analyzed using the appropriate software (SoftMax Pro 7, Molecular Devices Corporation, Orleans Drive, Sunnyvale, CA, USA, 2017). Freshly frozen plasma released from endogenous CGRP (extraction procedure) was used as a matrix for control samples (positive and negative controls) from the manufacturer and for preparing standards (S1 to S8) according to the manufacturer’s instructions. The patient samples in this procedure did not undergo the extraction process prior to the ELISA procedure, which is detailed in the manufacturer’s instructions.

Reading of results according to preliminary verification results was carried out at a wavelength of 405, 60 min after the end point of the ELISA procedure (after the addition of Ellman’s reagent). For CGRP values greater than 1000 pg/mL, the determination was repeated using a diluted sample with ELISA buffer. The manufacturer declares values ranging from 3 to 269 pg/mL (0.8 pmol/L to 71 pmol/L) as the range of values in healthy individuals. Elevated CGRP values are also found in patients undergoing hemodialysis, patients with exacerbated asthma, thyroid cancer patients, and pregnant women.

### 2.4. Statistical Analysis

Categorical data are presented with absolute and relative frequencies. Chi-squared and Fisher exact tests assessed categorical variable differences. Normality of distribution of continuous variables was tested with the Shapiro–Wilk test. Continuous data are described with the median and interquartile range. Differences in continuous variables between two independent groups were tested with the Mann–Whitney U test (Hodges–Lehmann median difference with corresponding 95% confidence interval). Differences in normally distributed numerical variables among more than two independent groups were tested with the Kruskal–Wallis test (post hoc Conover). The impact of CGRP on migraine probability was evaluated with logistic regression. ROC (Receiver Operating Characteristic) analysis was used to determine the optimal cutoff point, area under the curve (AUC), specificity, and sensitivity of the CGRP biomarker in predicting migraine probability. All *p*-values are two-sided. The level of significance was set at alpha = 0.05. Statistical analysis was conducted using MedCalc^®^ Statistical Software version 22.023 and SPSS 23.

## 3. Results

### 3.1. Study Population

Serum samples were collected from 66 children with migraine (40 MO, 12 MA, seven pMO, seven pMA), 59 children with tension-type headache (46 TTH, 13 pTTH) and 53 healthy controls. Samples from participants with primary headache were collected between headache episodes (interictally). The average age of the children with migraines was not significantly different from those of the subjects with tension-type headaches or the healthy controls. The demographic data and clinical characteristics of the study subjects are shown in Table 1.

In 48% of the participants, headaches started between the ages of 7 and 12 years, in 24%, at an age younger than 7 years, and the remaining participants were older than 12 years. There was no significant difference in age between the study groups. The duration of headaches was significantly longer in the migraine group compared to the tension-type group (median 25 months vs. 12 months). Although the migraine group had a longer duration of headache attacks (6 h vs. 4 h), it was not deemed significant. In terms of frequency of headaches, the tension-type group experienced more frequent headaches than the migraine group. Migraine with aura is usually viewed as a more complex type of migraine because it includes additional neurological symptoms and carries a higher risk of certain complications, such as ischemic stroke. Therefore, we also conducted a comparison between the migraine with aura and migraine without aura subgroups (Table 2). There were no statistically significant differences in comorbidities and previous episodic syndromes that may have been associated with migraine between the study groups.

### 3.2. Serum CGRP Levels in the Migraine Group, Tension-Type Group, and Healthy Controls

**Figure 1:**
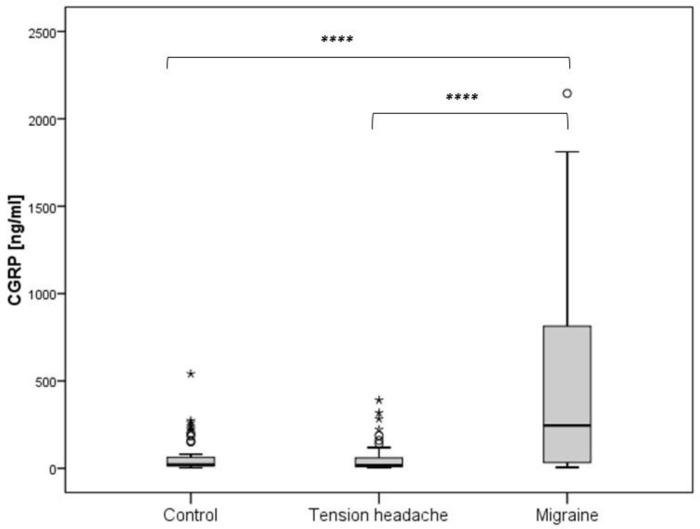
Difference in CGRP levels according to the subject groups (**** *p* < 0.05).

We examined the interictal serum levels of CGRP in the migraine group, tension-type group, and healthy controls. The migraine group had significantly higher levels of CGRP (CGRP(m): median 245.5, IQR 33.5–813.4) compared to the tension-type group (CGRP(t): median 17.3, IQR 9.8–60.8) and the healthy control group (CGRP(c): median 20.4, IQR 12.9–63.9). There was no significant difference in CGRP levels between the tension-type and control groups (Figure 1).

### 3.3. Plasma CGRP Levels in Pediatric Migraine Without Aura, Migraine with Aura, Probable Migraine Without Aura, and Probable Migraine with Aura

**Figure 2:**
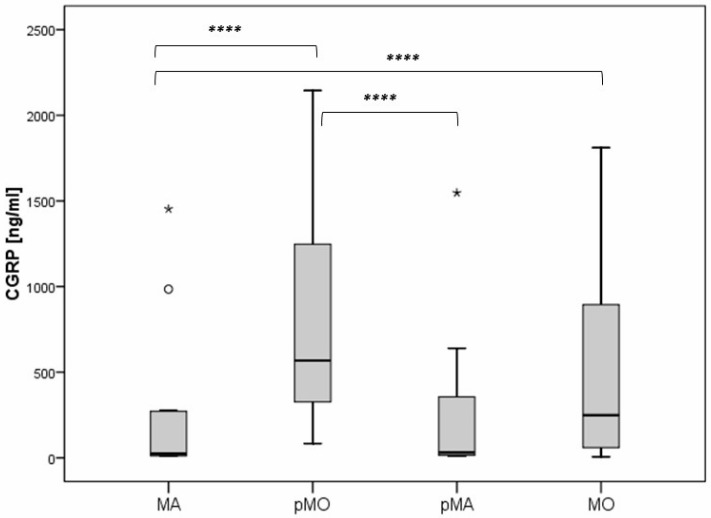
Difference in CGRP levels regarding the type of migraine headache. Legend: MA—migraine with aura; pMO—probable migraine without aura; pMA—probable migraine with aura; MO—migraine without aura (**** *p* < 0.05).

The CGRP levels were found to be significantly lower in the subjects with MA compared to MO and pMO (median 23.3 vs. 249.0 vs. 568.3), and the subjects with pMO had significantly higher CGRP levels compared to the subjects with pMA (median 568.3 vs. 30.4) (Figure 2).

**Figure 3:**
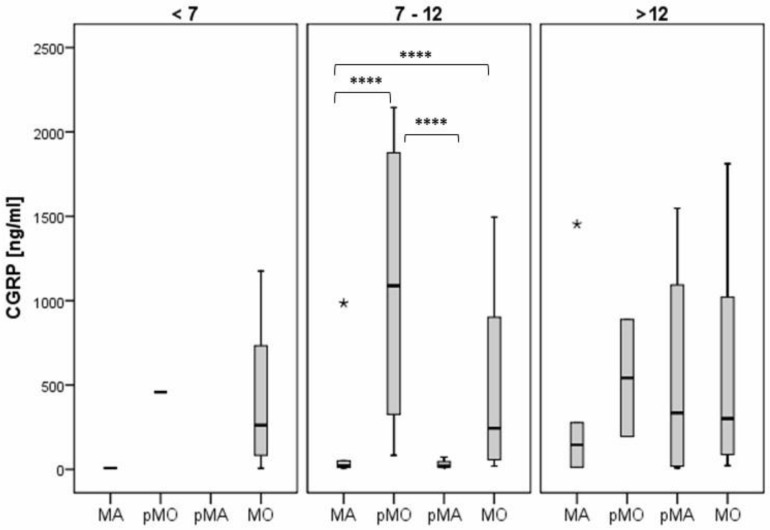
The difference in CGRP values according to the type of migraine headache. Legend: MA—migraine with aura; pMO—probable migraine without aura; pMA—probable migraine with aura; MO—migraine without aura (**** *p* < 0.05).

The CGRP levels were also significantly lower in the subjects with MA whose headaches began between the ages of 7 and 12 years compared to the subjects with MO (median 20.7 vs. 247.6), and similar results were found in the pMA/pMO subjects in the same age range (median 18.3 vs. 1087.7). There were no significant differences between the MA and MO subgroups for the subjects whose headaches began before age 7 and after age 12, and no significant differences were found between the pMA and pMO subgroups for subjects whose headaches began after age 12 (Figure 3).

### 3.4. The Correlation of CGRP with Clinical Characteristics and Association of CGRP and the Diagnosis of Pediatric Migraine

The univariate regression analysis revealed that the serum CGRP levels were significantly correlated only with visual (*p* = 0.009) and sensory (*p* = 0.04) aura (Table 3). We included clinical characteristics with a *p*-value of less than 0.2 in the multiple linear regression analysis, but the results indicated that none of the predictors showed a significant association with CGRP levels.

A logistic regression analysis was conducted to assess the impact of CGRP on the probability of migraine occurrence. The results indicate that CGRP is a significant predictor of migraine, with an odds ratio of 1.01. This means that individuals with higher CGRP levels have a 1.01 times increased likelihood of experiencing a migraine. Furthermore, CGRP accounts for between 28% (as per Cox and Snell R²) and 39% (according to Nagelkerke R²) of the variance in migraine occurrence, and it accurately predicts 78% of cases.

### 3.5. The Diagnostic Value of CGRP in Pediatric Migraine

When comparing the probability of migraine to that of the control group, CGRP emerged as a significant diagnostic marker for migraine (AUC = 0.779; sensitivity = 51.5; specificity = 94.3; *p* < 0.001), with a cut-off point of >241.5. It also served as a notable diagnostic indicator when differentiating migraines from tension-type headaches (AUC = 0.799; sensitivity = 56.1; specificity = 93.2; *p* < 0.001), with a cut-off point of >187.4.

**Figure 4:**
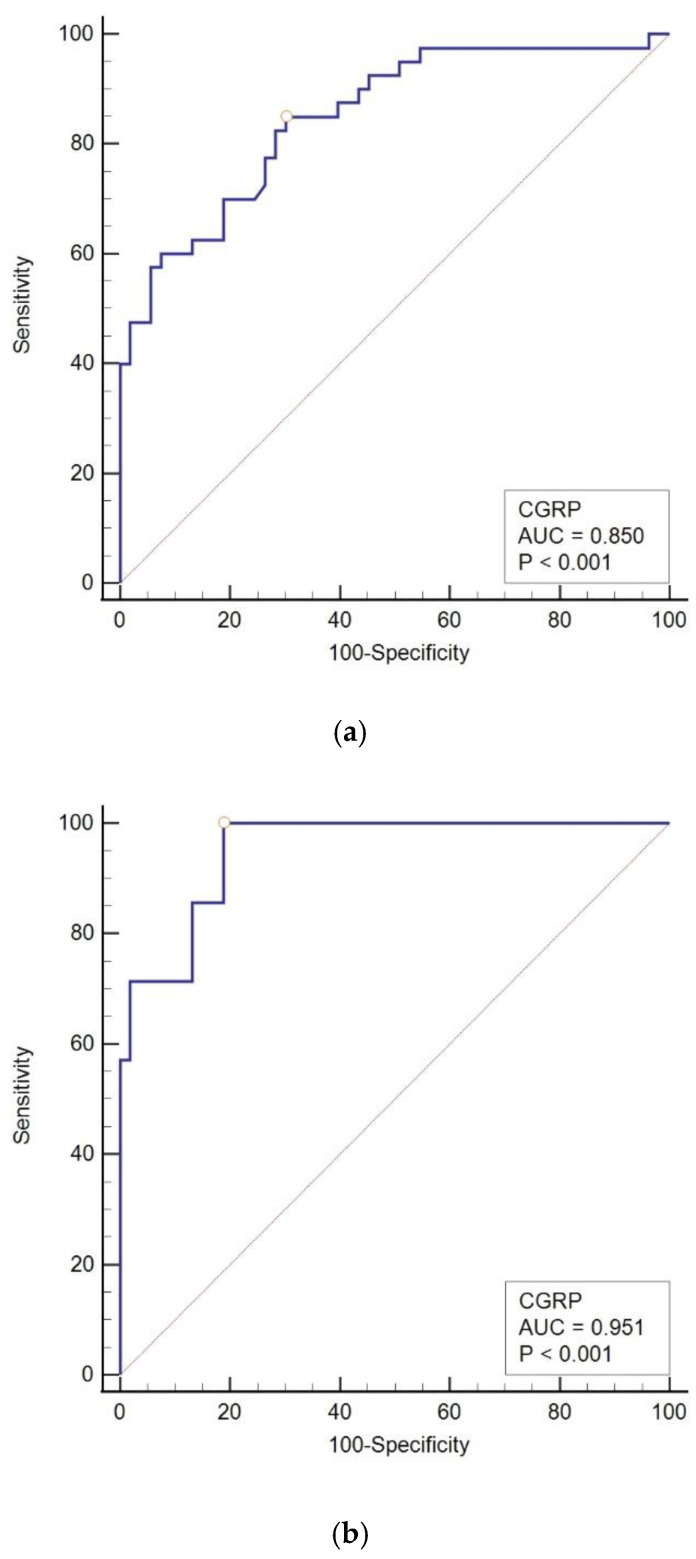

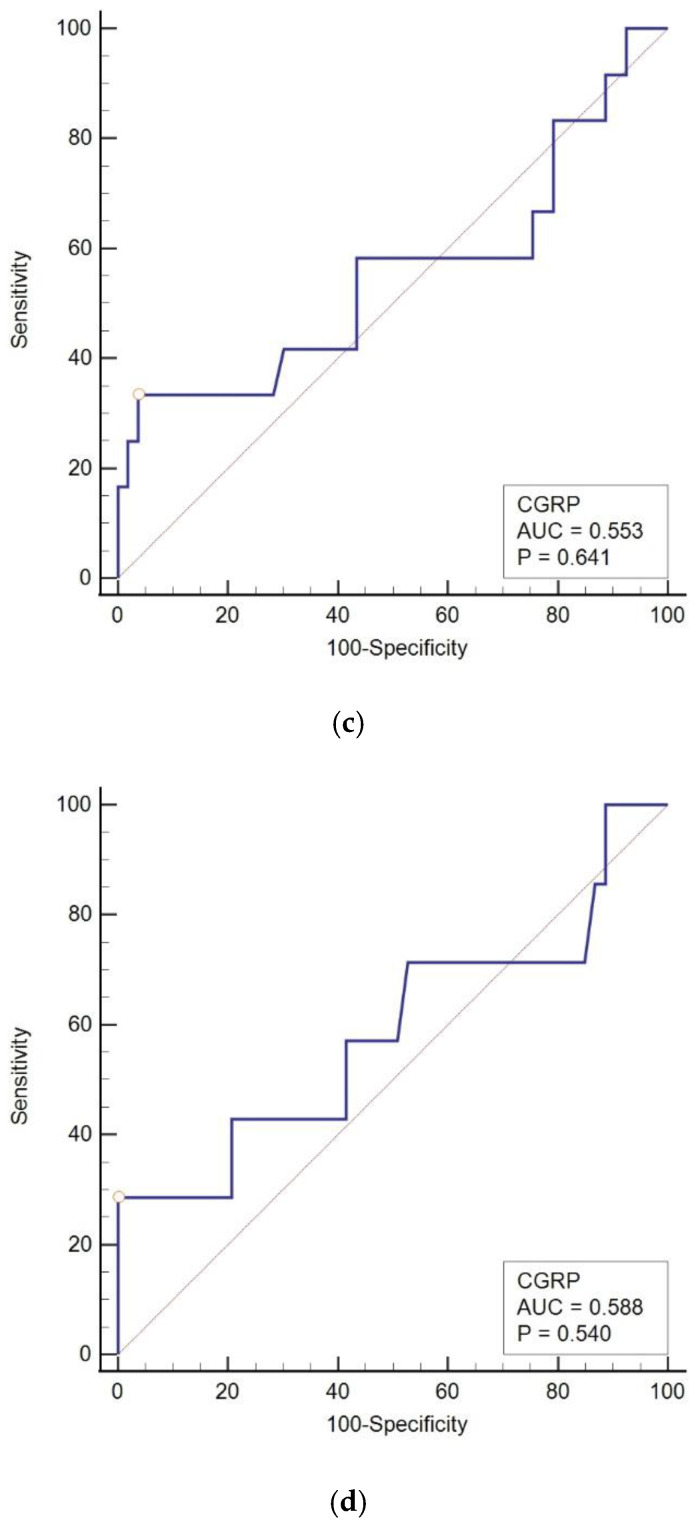
The ROC curve of CGRP in diagnosing pediatric migraine. (**a**) The ROC curve of CGRP in MO vs. control; (**b**) the ROC curve of CGRP in pMO vs. control; (**c**) the ROC curve of CGRP in MA vs. control; (**d**) the ROC curve of CGRP in pMA vs. control. MO: migraine without aura, pMO: probable migraine without aura, MA: migraine with aura, pMA: probable migraine with aura, AUC: area under the curve, CGRP: calcitonin-gene-related peptide.

In our analysis of the migraine subgroups (MO, MA, pMO, and pMA), CGRP was found to be a significant diagnostic indicator for both MO and pMO, but not for MA or pMA. When MO is considered independently from other migraine types, CGRP served as a significant diagnostic indicator for MO when compared to the tension-type and control groups. The results were even more pronounced than those observed in the entire migraine group, with a larger area under the curve (AUC 0.85 vs. 0.779) and with higher sensitivity (85% vs. 51.5%). Similarly, CGRP is a significant diagnostic indicator for pMO when compared to control and tension-type headache. However, the analysis of CGRP as a diagnostic indicator of MA did not show significance in comparison with the control or tension-type group of subjects, and the same applies for pMA (Figure 4).

## 4. Discussion

The results of our study indicate that interictal CGRP values are significantly higher in children with migraine than in children with tension-type headache or in healthy controls. The significance of this research lies in comparing migraine and tension-type headaches, given their often-overlapping characteristics, particularly in children under 7 years of age. To the best of our knowledge, this is the first study of interictal CGRP levels comparing children with migraine and tension-type headache. Two previously conducted studies compared interictal plasma CGRP levels in subjects with migraine and subjects with non-migraine headache, which included tension-type headache and secondary headaches [62,63].

Our subjects with MA had significantly lower CGRP levels than the subjects with MO. Moreover, there was no significant difference in interictal CGRP levels between the MA subgroup and healthy controls. This finding needs to be elucidated in future studies with larger sample sizes, yet the same outcome arises from the work of Al-Khazali et al., which showed that aura reduces the probability of migraine development induced by exogenous CGRP [68].

We found no significant difference in CGRP levels between the MO and MA subgroups in subjects with headache onset >12 years, but the difference was significant in the group in which the headache started at a younger age (7–12 years). Published studies comparing interictal CGRP levels in MO and MA in children showed no difference in interictal levels of CGRP between MO and MA [61,64]. In those studies, stratification for age was not performed, but the first study contained only adolescent subjects [61], and in the second study, subjects with MA had a significantly higher number of migraine attacks per month compared to our subjects [64]. Significant differences were observed in the CGRP values when stratified by aura type, particularly for visual and sensory auras. This suggests that the presence of certain types of aura may be associated with distinct CGRP levels, indicating potential differences in migraine pathophysiology based on aura presence.

In our study, we observed that the levels of CGRP remain consistent in individuals with MO regardless of their age at the onset of their headaches. However, in individuals with MA, the levels of plasma CGRP appear to be linked to their age. Research from animal studies indicates that CGRP is produced during the fetal period, with higher levels present in the fetus, before levels decrease as age progresses [69], which supports the idea of age-specific CGRP production. An age-related difference in TGVS response to capsaicin has been observed, especially in an adolescent and adult rat model, in which adolescent rats showed less reactivity, reinforcing the notion that age influences the presentation of disease due to variations in TGVS reactivity [70]. Thus, it is plausible that the pathophysiological processes underlying migraine, or migraine types, including CGRP production and response to CGRP, are influenced by age.

The absence of significant associations in the multiple regression analysis in this study may have several implications. It suggests that the clinical features of migraine examined in this study do not contribute significantly to CGRP levels. Previous research investigating the link between CGRP levels and clinical features of migraine, such as the frequency and duration of migraine attacks in children, has yielded mixed results. In the study conducted by Hanci et al., there was no correlation between CGRP levels and the frequency or duration of migraine attacks [65]. Conversely, Liu et al. identified a correlation between CGRP levels, attack duration (greater than 6 h), and the frequency of attacks (fewer than 15 attacks per month) [64]. A study on adult subjects found a correlation between CGRP levels and headache intensity and duration while the attack was occurring, with CGRP levels returning to baseline once the migraine episode ended [61]. A related finding was reported by Gupta et al., who observed a positive correlation between CGRP levels and the duration and frequency of headaches during attacks, although they did not confirm a higher interictal CGRP level in migraine patients compared to those in the control and tension-type headache groups [42]. The distinction in CGRP levels between episodic migraine and chronic migraine prompted researchers to conclude that the less frequent activation of the TGVS is the cause of lower CGRP levels in episodic migraine [71]. Studies that reported elevated interictal CGRP levels in episodic migraine were primarily conducted in tertiary care centers, suggesting that the included participants had a more severe form of migraine, characterized by increased frequency, pain intensity, and attack duration [57,58]. The continuous release of CGRP resulting from frequent headaches, as observed in CM, promotes the sensitization of central trigeminal neurons, and electrophysiological evidence indicates that the brain remains hyperexcitable between migraine episodes, making individuals more susceptible to subsequent migraine attacks [72]. Additionally, it has been demonstrated that chronic migraine patients have heightened sensitivity to exogenous CGRP, indicating that CGRP may also function as a modulator of nociceptive transmission within the trigeminal system [73]. It is plausible that the reactivity of the trigeminal vascular system differs in developmental stages compared to adulthood, and the triggers for its activation and the sensitization of central trigeminal neurons in children may vary.

Regarding the diagnostic value of CGRP in the study by Liu et al., CGRP was identified as a diagnostic indicator of migraine when compared to the control group. The ROC analysis showed an area under the curve of 0.869 (AUC), with a specificity of 76.62% and sensitivity of 85.53%, using a cutoff value above 94.29 pg/mL. However, the diagnosis of migraine in that study was clinical and not strictly based on the ICHD-3 criteria [64]. Our study achieved higher specificity but lower sensitivity, which is likely to have been due to the strict inclusion and exclusion criteria.

However, this study is limited by its small sample size, especially given that the migraine patients were categorized into four types, based on their migraine characteristics, and further subdivided into three groups according to the age of headache onset. The small number of subjects, particularly in the youngest age group, places a limitation on the study. Another limitation is that the data that were collected (frequency of migraines, duration of headaches, concomitant symptoms, etc.) depended on the memory of the participants and/or their parents, which may have resulted in bias in the gathered data.

Taking these limitations into account, it appears that interictal levels of CGRP are somewhat specific and sensitive to migraine in children, especially when only considering migraine without aura. Our findings only reflect a cross-sectional analysis of a specific period in the disease course, whereas epidemiological studies classify migraine as a lifelong condition [74]. Thus, a clearer understanding of CGRP levels would require longitudinal studies and follow-up with migraine patients throughout childhood, adolescence, and adulthood. Further research is necessary on younger children, especially considering the limited data on MA in this group, which should include subjects with episodic syndromes that may be related to migraine.

Given the rising prevalence of migraine, further research is crucial to elucidate the underlying mechanisms of this condition in children. Gaining this insight could assist in formulating preventive strategies to reduce the risk of chronic disease and related comorbidities. Ongoing monitoring of the increasing trends in the prevalence and incidence of primary headaches in childhood can aid in discovering ways to enhance headache health and lessen the long-term impacts of headache disorders worldwide.

## Figures and Tables

**Table 1:** Demographic data and clinical characteristics of the study subjects.

Characteristics	Migraine (*n* = 66)	Tension-Type Headache (*n* = 59)	Control (*n* = 53)	*p*-Value
Age (Years)	13 (9–14)	12 (9–16)	13 (6–18)	0.91 §
Gender (Male)	30 (45%)	20 (34%)	31 (58%)	0.03 †
Age at Onset				
<7	18 (27%)	12 (20%)		0.64 †
7–12	31 (47%)	29 (49%)		
>12	17 (26%)	18 (31%)		
Duration of Symptoms (Months)	25 (11–49)	12 (6–28)	-	0.005 *
Attack duration (Hours)	6 (3–12)	4 (2–8)	-	0.009 *
VAS				
Mild Pain	0	2 (3%)	-	<0.001 †
Moderate Pain	7 (11%)	47 (80%)	-	
Severe Pain	59 (89%)	10 (17%)	-	
Frequency of Episodes				
Rare Episodic	34 (52%)	11 (19%)	-	<0.001 †
Frequent Episodic	32 (48%)	48 (81%)	-	
Activity-Related Aggravation	62 (94%)	10 (17%)	-	<0.001 †
Aura	19 (29%)	-	-	-
Visual	13 (20%)	-	-	-
Sensory	10 (15%)	-	-	-
Speech	7 (11%)	-	-	-
Motor	1 (2%)	-	-	-
Brainstem	1 (2%)	-	-	-
Concomitant Symptoms				
Nausea	53 (80%)	8 (14%)	-	<0.001 †
Vomiting	42 (64%)	2 (3%)	-	<0.001 †
Photophobia	52 (79%)	14 (24%)	-	<0.001 †
Phonophobia	52 (79%)	6 (10%)	-	<0.001 †
Location of Pain				
Unilateral	25 (38%)	7 (12%)	-	0.001 †
Bilateral	41 (62%)	52 (88%)	-	-

Legend: * Statistical tests: * Mann–Whitney U test; † Fisher’s exact test; § Kruskal–Wallis test.

**Table 2:** Demographic data and clinical characteristics and comparison between subjects with migraine with aura and migraine without aura.

Characteristics	MA (*n* = 19)	MO (*n* = 47)	*p* Value
Age (Years)	14 (13–15)	12 (9–14)	0.001 *
Gender (M)	7 (37%)	23 (49%)	0.37 †
Course (Months)	12 (5–48)	26 (12–50)	0.04 *
Duration of Attacks (Hours)	6 (3–10)	4 (3–12)	0.90 *
VAS (Visual Analog Scale)			
Moderate Pain	1 (5)	6 (13)	0.66 †
Severe Pain	18 (95)	41 (87)	
Frequency			
Rare Episodic	14 (74)	20 (43)	0.02 †
Frequent Episodic	5 (26)	27 (57)	
Activity-Related Aggravation	19 (100)	43 (92)	0.32 †
Associated Symptoms	9 (47)	25 (88)	0.67 †

Legend: * Mann–Whitney U test; † Fisher’s exact test; bold values denote statistical significance at the *p* < 0.05 level; MA—migraine with aura; MO—migraine without aura.

**Table 3:** Univariate analysis of the correlation between CGRP and clinical characteristics.

Characteristic	CGRP Median (IQR)	*p*-Value
Age (years)		
0–7	250 (24.8–665.7)	0.10 *
7–12	276.7 (763.0–1030.8)	
12–18	74.8 (19.6–646.5)	
Sex		
Male	274.8 (56.6–686.9)	0.69 †
Female	221.3 (23.3–932.5)	
Course (months)		
0–6	134.2 (24.4–912.1)	0.95 *
6–12	283.6 (54.5–990.5)	
12–18	371.3 (56.6–813.4)	
18–24	75.8 (56.9–559.6)	
>24	245.7 (23.3–686.9)	
Attack duration (hours)		
0–2	568.3 (91.3–1036.6)	0.21 *
2–4	88.3 (21.3–515.2)	
4–6	73.3 (12–199.3)	
>6	288.7 (50.4–976.3)	
Frequency		
Rare episodic	96.1 (49.2–639.1)	0.21 †
Frequent episodic	303.2 (29.2–983.4)	
VAS (Visual Analog Scale)		
Moderate pain	617.6 (112.1–1404.7)	0.17 †
Severe pain	232.2 (31.2–771.7)	
Activity-related aggravation		
Yes	247.8 (49.2–813.4)	0.47 †
No	40.1 (18.1–834.2)	
Aura		
Visual	23.2 (11.6–359.6)	0.009 †
Sensory	31.2 (9–276.7)	0.04 †
Speech	266.4 (21.2–548.5)	0.63 †
Motor	8.7 (*n* = 1)	-
Brainstem	266.4 (*n* = 1)	-
Concomitant symptoms		
Nausea	199.3 (32.7–671.0)	0.34 †
Vomiting	149.6 (24.8–686.9)	0.35 †
Photophobia	247.8 (23.3–806.7)	0.65 †
Phonophobia	247.8 (23.3–806.7)	0.65 †
Pain localization		
Unilateral	199.3 (29.0–653.2)	0.57 *
Bilateral	247.6 (56.1–978.5)	

Legend: IQR—interquartile range; * Kruskal–Wallis test; † Mann–Whitney U test.

## Data Availability

The datasets presented in this article are not readily available because they are part of a larger study and analyses are still ongoing. If requested, the corresponding author can send data directly by email.
